# Self-Control of Haptic Assistance for Motor Learning: Influences of Frequency and Opinion of Utility

**DOI:** 10.3389/fpsyg.2017.02082

**Published:** 2017-12-04

**Authors:** Camille K. Williams, Victrine Tseung, Heather Carnahan

**Affiliations:** ^1^Rehabilitation Sciences Institute, University of Toronto, Toronto, ON, Canada; ^2^School of Human Kinetics and Recreation, Memorial University of Newfoundland, St. John’s, NL, Canada

**Keywords:** guidance, concurrent feedback, path-following, thematic analysis, learner-controlled, autonomy support, training, tracing

## Abstract

Studies of self-controlled practice have shown benefits when learners controlled feedback schedule, use of assistive devices and task difficulty, with benefits attributed to information processing and motivational advantages of self-control. Although haptic assistance serves as feedback, aids task performance and modifies task difficulty, researchers have yet to explore whether self-control over haptic assistance could be beneficial for learning. We explored whether self-control of haptic assistance would be beneficial for learning a tracing task. Self-controlled participants selected practice blocks on which they would receive haptic assistance, while participants in a yoked group received haptic assistance on blocks determined by a matched self-controlled participant. We inferred learning from performance on retention tests without haptic assistance. From qualitative analysis of open-ended questions related to rationales for/experiences of the haptic assistance that was chosen/provided, themes emerged regarding participants’ views of the utility of haptic assistance for performance and learning. Results showed that learning was directly impacted by the frequency of haptic assistance for self-controlled participants only and view of haptic assistance. Furthermore, self-controlled participants’ views were significantly associated with their requested haptic assistance frequency. We discuss these findings as further support for the beneficial role of self-controlled practice for motor learning.

## Introduction

One criticism of studies in motor learning is the overemphasis on the role of the teacher to direct the learning experience while the role of the learner has been minimized (Janelle et al., 1995). Recently, more researchers have begun to explore this notion by distributing the responsibilities for learning between experimenter/instructor and participant/learner. Such studies have consistently shown that there are learning benefits when learners are given the opportunity to control aspects of the practice environment such as feedback schedule (Janelle et al., 1995, 1997; Chiviacowsky and Wulf, 2002; Aiken et al., 2012; Fairbrother et al., 2012), use of assistive devices (Wulf and Toole, 1999; Hartman, 2007), amount of practice (Lessa and Chiviacowsky, 2015), and task difficulty (Andrieux et al., 2012, 2015). These benefits are typically in relation to a “yoked” group of participants whereby each yoked participant is matched to a self-controlled participant and therefore receives the same choice-based element of practice as that of self-controlled participant. Although numerous studies have demonstrated the advantage of self-controlled practice for learning (for reviews, see Wulf, 2007; Sanli et al., 2013), there is little empirical evidence to unequivocally support the reasons or mechanisms for this advantage (Sanli et al., 2013). Most authors have cited either motivational or cognitive (i.e., related to information processing) processes for the observed benefits but an antagonistic relationship between the two has also been suggested (Bund and Wiemeyer, 2004).

The importance of motivational processes can be understood through self-determination theory which posits that autonomy, competence and relatedness are fundamental psychological needs underlying intrinsic motivation for goal-directed behavior (Deci and Ryan, 2000). As such, supporting intrinsic motivation and autonomy (e.g., by providing choices) can lead to improved performance and learning^1^ (Patall et al., 2008). Autonomy support has slightly different definitions across domains but generally refers to the actions and sentiments of one person provided to enhance another person’s subjective experience of autonomy (Su and Reeve, 2011). An autonomy supportive environment can be achieved through a combination of five interpersonal conditions: providing meaningful rationales, acknowledging negative feelings, using non-controlling language, offering choices, and nurturing inner motivational resources. However, it appears that researchers in the motor learning domain have focused on offering choices (particularly about feedback scheduling) as the primary method of supporting participants’ autonomy (Sanli et al., 2013). A meta-analysis (not limited to the motor learning domain), that examined the effect of choice on intrinsic motivation and related outcomes, found a positive and significant effect of choice on intrinsic motivation, perceived competence, effort and task performance (Patall et al., 2008).

In the motor learning domain, findings have been mixed with respect to a clear benefit of self-controlled practice for intrinsic motivation and related outcomes such as self-efficacy, perceived competence, perceived autonomy, and affect. Several studies have found differences between self-controlled/autonomy-supported groups and yoked/autonomy-restricted groups for measures related to motivation (Bund and Wiemeyer, 2004; Chiviacowsky et al., 2012a,b; Chiviacowsky, 2014; Hooyman et al., 2014; Wulf et al., 2014, 2015; Grand et al., 2015; Lemos et al., 2017), but a few studies have failed to find these differences (Ste-Marie et al., 2015; Carter and Ste-Marie, 2017). The literature also show that participants use their autonomy to enhance perceived competence by requesting feedback after perceived good trials (Chiviacowsky and Wulf, 2002; Fairbrother et al., 2012). Notably, while most studies have employed autonomy support around task-relevant activities or components of the training environment, some studies have specifically shown that making task-irrelevant or incidental choices can also have beneficial effects for motivation and/or motor learning (Wulf and Adams, 2014; Wulf et al., 2014; Lewthwaite et al., 2015). In fact, results from Patall et al.’s (2008) meta-analysis showed that instructionally irrelevant choices had the greatest positive impact on intrinsic motivation. However, at least one motor learning study has failed to find an effect of task-irrelevant choices when specifically compared to task-relevant choices (Carter and Ste-Marie, 2017).

In contrast to these explorations of the motivational impacts of self-control, some motor learning researchers have focused on the potential information processing benefits of autonomy supportive training environments (Wulf, 2007; Sanli et al., 2013). In particular, studies have shown that choosing feedback after (vs. before) trials (Chiviacowsky and Wulf, 2005; Carter et al., 2014) is required to achieve the learning benefits of self-controlled feedback and that self-control of feedback leads to improved error estimation abilities (Chiviacowsky and Wulf, 2005; Carter et al., 2014) as well as enhanced feedback processing (Grand et al., 2015). Proposed reasons for enhanced information processing include that these participants are able to request feedback when they think it will be useful or they are inherently more engaged in the learning process and intrinsically motivated to learn (Sanli et al., 2013; Grand et al., 2015). Importantly, however, Grand et al.’s (2015) finding that intrinsic motivation and feedback processing together predicted a measure of motor learning strongly suggests that motivational and cognitive strategies could be inter-related. Self-controlled participants’ selection of feedback after good trials coupled with enhanced feedback processing in relation to yoked participants, could be interpreted as self-controlled participants processing more information specifically to confirm good trials, i.e., to enhance perceived competence – a motivational strategy. However, this could also be interpreted as a cognitive/informational strategy since feedback after good trials better enabled self-control participants to learn how best to fine-tune the forces required for the task (Grand et al., 2015).

Despite the well-documented benefits of self-controlled learning and the exciting mechanistic implications, researchers have yet to explore whether self-control could be beneficial for motor learning in the haptic training domain. Haptic training can be defined as the use of hardware and software that manipulate computer-controlled sensations of touch (tactile and/or proprioceptive) for the purpose of improving skill or ability to perform a task (Williams and Carnahan, 2014). The most well-studied form of haptic training is assistive haptic feedback, also known as haptic assistance, haptic guidance or robot assistance (Williams and Carnahan, 2014). This form of training concurrently provides feedback about performance while minimizing errors to assist in task performance (see Heuer and Lüttgen, 2015 for a review). Importantly, there is one major difference between the paradigms of haptic training and self-controlled learning that might modulate the effects of self-controlled haptic feedback on motor learning. Specifically, studies of self-controlled practice typically employ terminal feedback as opposed to the concurrent feedback provided by haptic assistance. This is particularly relevant because researchers have consistently found that self-controlled learners tend to prefer receiving feedback after perceived good trials (e.g., Chiviacowsky and Wulf, 2002; Grand et al., 2015; Laughlin et al., 2015) or equally after perceived good and bad trials (Carter and Patterson, 2012), and previous research using terminal feedback suggests that self-controlled feedback is effective when it is based on the learner’s performance, i.e., the decision is made after the trial (Chiviacowsky and Wulf, 2005; Carter et al., 2014). However, with concurrent feedback, the learner is unable to choose feedback *after* a trial; participants must decide whether to have feedback either *before* or *during* a trial. To date, there have been only a few studies utilizing self-controlled concurrent feedback and they utilized visual (not haptic) feedback. Notably, a pair of studies led by the same author and both published in 2009, investigated self-controlled concurrent feedback in two very different practice contexts. In the first study, the task was a complex perceptual-motor door crossing task whereby participants had to adjust their walking speed on a treadmill such that they would cross a virtual door threshold when the opening between doors was the widest (Huet et al., 2009a). The concurrent feedback represented the error that would result if participants maintained their current walking speed. In the second study, the task was landing a virtual aircraft in a fixed-base flight simulator with feedback representing augmented information about the aircraft’s current glide slope, that is, the angle of approach to the runway (Huet et al., 2009b). Participants in both studies could choose to display visual concurrent feedback about performance at multiple time-points during a trial. Results from both studies showed that the self-controlled feedback participants outperformed their yoked counterparts. Interestingly, participants in the door crossing study opted for a faded feedback schedule (i.e., they reduced their requests for feedback over the course of skill acquisition) (Huet et al., 2009a), while participants in the virtual aircraft landing study did not fade their feedback but adapted the functional role of feedback requests from discovery to confirmation of the relationship between relevant sources of information (Huet et al., 2009b).

It is also useful to consider that, in addition to providing concurrent feedback, haptic assistance also provides physical assistance to complete the task, in part by altering the task difficulty. Study of the latter function (assistive/difficulty-reducing) is likely necessary to fully understand how these two practice features (haptic assistance and self-control) interact; especially because, in contrast to choosing feedback, choices about assistive devices and task difficulty are made *before* a trial. Studies exploring learning benefits of self-controlled physically assistive devices offered their self-controlled participants the use of poles for learning to use a ski simulator (Wulf and Toole, 1999; Wulf et al., 2001) or stabilometer (Hartman, 2007; Chiviacowsky et al., 2012b). These studies found that the self-controlled assistive device participants adopted a fading schedule for using the poles and performed better and/or more efficiently in retention when compared to their yoked counterparts. Additionally, Hartman (2007) reported that the self-controlled assistive device group asked for the pole when trying new movement strategies. However, studies have shown that while provision of assistive devices can facilitate exploration of movement strategies, it can also prevent this exploration by keeping participants “on-target” (Wulf and Shea, 2002). Studies of task difficulty have also shown that participants who could control task difficulty in skill acquisition, showed learning benefits in relation to those who experienced externally imposed task difficulty (Williges and Williges, 1977; Andrieux et al., 2012, 2015) and these benefits were enhanced when choice was only available in the first half of practice (Andrieux et al., 2015). The strategy of these self-controlled task difficulty participants was to continually challenge themselves throughout practice, moving toward the level of difficulty of the retention test.

In sum, it is unknown whether self-control of haptic assistance, which serves both feedback and assistive functions, will be beneficial for learning a self-paced curve-tracing task, how participants will choose to schedule it or for what functional role(s) participants will use it. As such, the present study had three Aims: (1) to explore how self-controlled participants choose to schedule haptic assistance; (2) to determine whether self-control of haptic assistance during skill acquisition is beneficial for learning; and (3) to explore the rationales and opinions held by both self-controlled and yoked participants with respect to their chosen or externally imposed haptic assistance schedules and whether these are related to motor learning. Data were collected to address all these Aims under one experimental protocol. However, to enhance clarity of the manuscript, we separated our reporting of the methods and results into two phases: Phase One addressed Aims 1 and 2, while Aim 3 was addressed in Phase Two. Motor learning was assessed using a transfer design. Specifically, skill acquisition, under various conditions defined by presence and choice of augmented haptic feedback, was followed by retention tests without any augmented feedback (Salmoni et al., 1984). This design allows for a distinction between the immediate and transient performance effects seen during and immediately after practice, and learning effects that represent more stable and permanent changes in ability. We hypothesized that, in accordance with studies of self-control of perceived assistive devices (e.g., Hartman, 2007) and concurrent feedback (e.g., Huet et al., 2009b), self-controlled participants, selecting haptic assistance before skill acquisition trials, would display a learning advantage. Additionally, we anticipated that differences in rationales, opinions or strategies related to the use of haptic assistance would also have some bearing on motor learning (Carter et al., 2016).

## Phase One

### Methods

#### Participants

The University of Toronto Health Sciences Research Ethics Board approved the protocol. We recruited 45 community-living, university-affiliated adults with normal or corrected-to-normal vision, who reported no current neurological impairments: 35 women and 10 men (*M* = 26.3 years, *SD* = 7.2); 5 were classified as left-hand dominant based on the online version of the Edinburgh Handedness Inventory^2^ (Oldfield, 1971). All participants were naïve to the specific purposes of the experiments and gave written voluntary informed consent prior to participation, in accordance with the guidelines set out by the 1964 Declaration of Helsinki. Participants received gift cards valued at CAD$15 as compensation for their time.

#### Apparatus and Task

**FIGURE 1:**
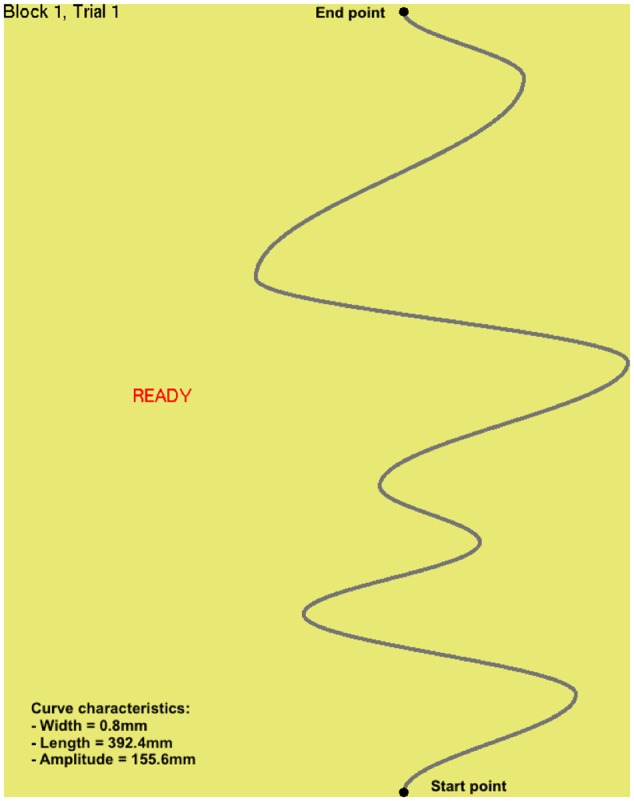
Cropped and annotated screen-shot showing the target curve along with curve characteristics. This figure is an edited version of one previously published (Williams et al., 2016).

The apparatus consisted of a tabletop haptic device (SensAble Phantom Omni, currently Geomagic Touch; Rock Hill, SC, United States) and standard computer monitor operated via a custom software program as previously reported (Williams et al., 2016), with a few noted exceptions, and we refer interested readers to that article for details omitted here. The computer monitor used in the present study was a Dell UltraSharp^TM^ 1703FP and the resolution resulted in a visual gain of 1.38 between displacements on the monitor and movements of the haptic device in space. The device was programmed such that it was possible to deliver assistive feedback forces as users, seated at a desk, attempted to trace a curve (**Figure 1**) by manipulating the device’s stylus with their non-dominant arm. The feedback forces were such that, if a user deviated from the target curve, the device delivered a linearly increasing force directed toward the curve. The position of the stylus was represented onscreen by a circular cursor.

#### Procedure

There were three skill acquisition conditions: (i) a control (CN) condition with no manipulation of tracing errors using the “none” haptic feedback mode; (ii) a self-controlled (SC) error minimization condition using the “spring assistance” mode of haptic feedback; and (iii) a yoked (YK) error minimization condition using the “spring assistance” mode. Using the Research Randomizer website^3^, participants were randomized to one of these three conditions for practice of the task, with the constraints that group assignments were equal in number (i.e., 15 participants in each group) and a self-controlled participant must precede its yoked counterpart.

After introductory explanation of the task, participants were allowed three familiarization trials with a curve different than the one to be learned. Once participants were comfortable with the device and the task, they did a pretest consisting of five trials of the task: once the target curve appeared on the computer screen, participants started the trial by moving the cursor to the start-point near the bottom of the screen and ended the trial by moving the cursor to the end-point near the top of the screen. The target curve and the cursor were visible for the entire trial and participants were instructed to trace the curve as quickly and as accurately as possible. Solely the cursor and the target curve were visible throughout these pretest trials and participants did not receive any augmented haptic feedback or other feedback about performance.

Following this was the skill acquisition/practice phase: 60 trials organized as 12 blocks (5 trials/block). Before beginning, all participants were informed that, like the pretest, all tests following practice would not contain any haptic or visual feedback about performance. The experimenter then explained, per their group assignment, what they should expect with respect to feedback (haptic and otherwise) during the practice phase. For participants in the CN group, the trial would proceed in the same way as the pretest trials. However, at the end of each trial, terminal feedback regarding the tracing accuracy (a red trace of their movement superimposed over the target curve) and movement time (a numerical value displayed in seconds to the nearest decisecond) were provided onscreen. For SC participants, the experimenter asked whether they wanted to have haptic assistance (termed “guidance”) prior to each block of trials. If they opted to have haptic assistance, then each trial in the block was accompanied by haptic assistance in accordance with their performance. They were informed that they could choose to have as many or as few guided blocks of practice as they liked. Each YK participant was matched to a participant in the SC group and was simply informed whether each upcoming block of trials would have haptic assistance available. SC and YK participants also received the other forms of terminal feedback regarding tracing accuracy and movement time that were available to participants in the CN group.

Ten minutes after the end of practice (after completing a pen-and-paper questionnaire – see Phase Two below for details), participants completed an immediate retention test which was identical to the pretest. After approximately 1 day (*M* = 1.2, *SD* = 0.4), participants returned for a delayed retention test (identical to the immediate retention test).

#### Outcome Measures and Data Analysis

For each SC participant, we noted the blocks on which haptic assistance was requested and employed a paired samples *t*-test to compare the number of guided blocks in the first and second halves of practice. The primary dependent variable was Speed Accuracy Cost Function (with units mm s), a measure of performance efficiency: Cost function = Tracing error × Movement time (Culmer et al., 2009; Raw et al., 2012; Williams et al., 2016). A large cost function indicated less efficient and overall, poorer, task performance. Tracing error for each trial, measured in mm, was calculated as:

Tracing error=1N∑i = 1N|e[i]|

where *N* is the total number of samples in a trial and *e*[*i*] is the distance between the cursor and the next untraced portion of the target curve on sample *i*. Movement time for each trial was measured as the time (in seconds to the nearest millisecond) from when the cursor was moved to the start-point, to when the cursor was moved to the end-point.

Cost function values were averaged over each block of 5 trials to provide 12 data points for skill acquisition and three data points for tests of skill (pretest, immediate and delayed retention). We first conducted a one-way between-subject ANOVA, to compare the effect of practice group on pretest cost function values. There were no significant differences between groups [*M* = 26.23 mm s, *SD* = 7.11; *F*(2,40) = 1.74, *p* = 0.189, $\eta_{p}^{2}$ = 0.08] so we proceeded with the analyses described below and data from the pretest will not be discussed further. We conducted two mixed ANOVAs (3 group × 12 block in acquisition; 3 group × 2 test in retention) with repeated measures on each of the respective last factors. Because we expected that performance would improve over the course of acquisition (i.e., cost function would decrease), main effects or interactions involving block in acquisition were explored using contrasts with the first block as the reference category. When Mauchly’s test indicated that the assumption of sphericity had been violated for repeated measures factors, Greenhouse-Geisser corrections were applied (all 𝜀 < 0.75) and adjusted degrees of freedom were reported to the nearest decimal. When simple main effects analyses were used to explore significant interactions, Bonferroni corrected *p*-values were reported. Effects for all analyses were considered statistically significant at *p* < 0.05 and effect sizes associated with *F*-tests were estimated using partial eta squared values ($\eta_{p}^{2}$). Analyses of data from acquisition and immediate retention demonstrate the immediate effects of our practice conditions, both during and shortly following practice. However, learning effects were inferred based on analyses of delayed retention data.

### Results

Data from two participants (one from each of the SC and CN groups) were excluded from quantitative analyses of performance in acquisition and retention data because these participants struggled with correct use of the device. Their observed difficulties were also evident as elevated means and standard deviations of speed accuracy cost function throughout practice and retention.

#### Requests for Haptic Assistance

The range for requests of haptic assistance was large with a minimum of 0 guided blocks and maximum of 11 guided blocks (1 participant requested each of the minimum and maximum values, respectively). On average, SC participants requested haptic assistance for 36.3% of skill acquisition blocks. Haptic assistance was requested for *M* = 2.0 blocks (*SD* = 1.5) in the first half of practice and *M* = 2.4 blocks (*SD* = 2.3) in the second half of practice. A comparison of requests in each half indicated no significant difference, *t*(13) = -0.717, *p* = 0.486.

#### Performance during Skill Acquisition

**FIGURE 2:**
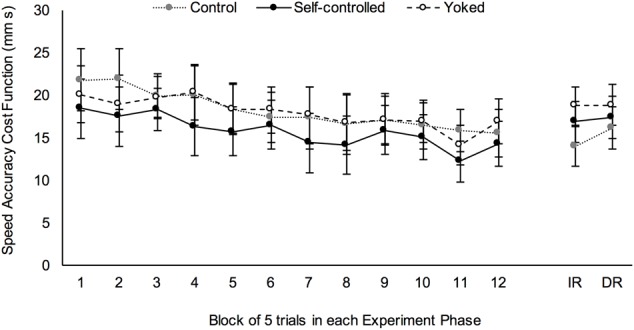
Overall performance efficiency (as measured by the speed accuracy cost function) for each group of participants in each phase of the experiment. Blocks of acquisition are numbered 1 through 12 and retention tests are IR (immediate retention) and DR (delayed retention). Error bars are 95% CI of the mean.

**FIGURE 3:**
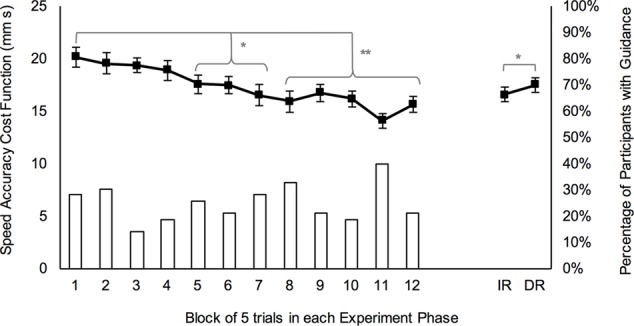
Overall performance efficiency (as measured by the speed accuracy cost function; line graph with SE bars) and percentage of all participants performing with haptic assistance (bar graph) for each phase of the experiment. Blocks of acquisition are numbered 1 through 12 and retention tests are IR (immediate retention) and DR (delayed retention). ^∗^*p* < 0.05, ^∗∗^*p* < 0.001.

Performance of each group throughout skill acquisition is shown in **Figure 2**. Analysis of this data showed that Mauchly’s test of sphericity was significant, χ^2^(65) = 168.2, *p* < 0.001, so degrees of freedom were corrected using Greenhouse-Geisser estimates. There was a main effect of block, *F*(6.3,246.2) = 9.2, *p* < 0.001, $\eta_{p}^{2}$ = 0.19 (**Figure 3** line graph, left side) and contrasts revealed that cost function on acquisition block 1 was significantly higher than cost function on blocks 5–7 (all *p* < 0.05) and blocks 8–12 (all *p* < 0.001). However, there was no effect of practice group, *F*(2,39) = 1.4, *p* = 0.248, $\eta_{p}^{2}$ = 0.07.

#### Performance on Retention Tests

There was a main effect of test, *F*(1,40) = 4.2, *p* = 0.048, $\eta_{p}^{2}$ = 0.09, whereby performance on the immediate retention test was significantly better than performance on the delayed retention test, *M*_diff_ = -0.93 mm s, 95%CI [-1.85, -0.01] (**Figure 3** line graph, right side). Meanwhile, the effect of practice group was not statistically significant, *F*(2,40) = 2.8, *p* = 0.074, $\eta_{p}^{2}$ = 0.12 (see **Figure 2** for comparison of performance between groups).

Because it is known that frequent feedback during skill acquisition can negatively impact learning (Salmoni et al., 1984; Park et al., 2000), we conducted an additional analysis of retention performance for the experimental groups (SC and YK) to determine if this was the case here. The distribution of requests for haptic assistance was such that sixty four percent of SC participants (*n* = 9) requested 4 or fewer guided blocks – these participants were classified as Low Frequency; the remaining 36% of participants (*n* = 5) requested 6 or more guided blocks and were classified as High Frequency. Because the sample size for each frequency category were different and unequal sample sizes can affect the accuracy of the *F*-test, for each experimental group separately, we conducted Mann–Whitney *U* tests to compare the learning outcomes of the low and high frequency participants on each retention test (immediate and delayed). Since we have reason to believe that the high frequency participants will have worse outcomes (i.e., greater SACF) than low frequency participants, we used one-tailed probability values to explicitly test this hypothesis. Additionally, due to relatively small samples, we opted for exact rather than asymptotic calculations of the test statistics. We estimated the effect size, *r* as the ratio of the *z*-score to the square root of the total sample size on which the test-statistic was based (Field, 2009, p. 550).

**FIGURE 4:**
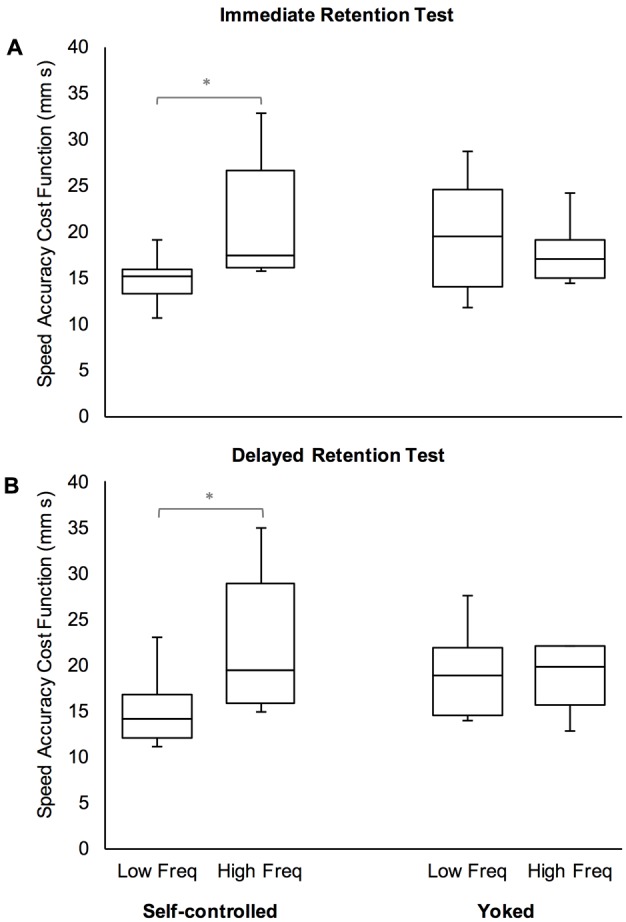
Box and whisker plots of performance efficiency (as measured by the speed accuracy cost function) for immediate **(A)** and delayed **(B)** retention tests, by experimental group and frequency of haptic assistance during skill acquisition. “Low Freq” refers to participants who practiced with a low frequency of haptic assistance (four or fewer blocks) while “High Freq” refers to participants who practiced with a high frequency of haptic assistance (more than four blocks). ^∗^*p* < 0.05.

For the SC participants, the results showed that low frequency practice produced significantly better outcomes than high frequency practice, for both the immediate, *U* = 5.00, *z* = -2.33, *p* = 0.009, *r* = -0.62 and delayed retention tests, *U* = 8.00, *z* = -1.3, *p* = 0.030, *r* = 0.52 (**Figure 4**, left sides). However, for YK participants, there was no difference between the low and high frequency sub-groups for the immediate, *U* = 22.00, *z* = -0.59, *p* = 0.303, *r* = -0.145, or delayed retention test, *U* = 26.00, *z* = -0.12, *p* = 0.477, *r* = -0.03 (**Figure 4**, right sides).

## Phase Two

### Methods

All methods described here were part of the same protocol described in Phase One and approved by The University of Toronto Health Sciences Research Ethics Board.

#### Participants and Procedures

Participants were those from the self-controlled and yoked groups described in Phase One above. At the end of skill acquisition described in Phase One, participants were asked to complete a pen and paper questionnaire regarding their practice experience. The questions (**Table 1**) were based on questionnaires employed in previous studies (Laughlin et al., 2015; Carter et al., 2016).

**Table 1:** Open-ended questions asked of self-controlled and yoked participants at the end of skill acquisition.

	Self-controlled group	Yoked group
Thinking about the first/last six blocks of trials:	When did you ask for haptic guidance? Why did you ask for haptic guidance at these times?	Did you receive haptic guidance at the right times for you? If so, why were these times right? If not, when would you have preferred to receive haptic guidance? Why would these times have been better?
Reflecting on the entire session:	What changes, if any, did you notice in your approach, thinking or process over the course of practice? Is there anything else you want us to know about your experience?	

Participants were allowed up to 10 min to complete the questionnaire and the experimenter answered any clarifying questions. Upon completion of the questionnaire, the experimenter quickly reviewed and asked for clarification on responses deemed illegible or the likely result of a misunderstanding by the participant. For example, in response to the question “Did you receive it [haptic assistance] at the right times for you?” addressed to YK participants, some interpreted the question as a query regarding whether haptic assistance *within a trial* was appropriately applied. In such cases, the experimenter explained that the question instead referred to the placement of guided blocks within the practice period.

#### Qualitative Data Analysis

Data consisting of text responses to the open-ended questions were analyzed using an inductive thematic analysis (Braun and Clarke, 2006). The primary analyst (CKW) transcribed each response then reviewed all responses repeatedly to get a sense of the whole. Subsequently, considering the response to each question for each participant separately, the analyst tagged each individual idea represented in the text with a code. For example, in response to the question “When did you ask for haptic guidance?”, a SC participant replied “When frustrated w/doing task w/o guidance – difficult to do w/left hand; When performance w/o guidance was not too great.” This entire excerpt was tagged with the codes “Chose guidance to improve performance” and “Chose guidance to make the task easier.” Each excerpt/response was tagged with as many codes as required to capture all the ideas present. Codes were kept organized according to the questions on the questionnaire to keep track of whether they applied to a SC or YK participant and whether they applied to the first or second half of practice. Once the analyst was satisfied that all ideas in the data had been captured by codes, the codes were examined for similarities within and across questions. Similar codes within questions were consolidated and similar codes across questions were renamed to be represented by the same code. Subsequently, all codes were reviewed multiple times, paying attention to any potential differences between choice-related practice groups (SC and YK). However, when considering the data for the SC and YK groups separately, themes emerging from each data set were very similar. As such, data from these groups were combined. Finally, in an iterative process, related codes were grouped into themes that applied across both groups of participants. Representative excerpts for each theme were noted.

We employed analyst triangulation whereby a second analyst (VT) independently reviewed the codes and themes developed by the primary analyst. At the time of analysis, VT was a doctoral candidate who employed qualitative methods in a research area unrelated to motor learning. The purpose of analyst triangulation is to produce multiple ways of seeing the data and facilitate discussions to ensure that a rich, robust and comprehensive description of the data was represented in the final set of themes (Yardley, 2015).

#### Quantitative Data Analysis

Once the themes were finalized, the primary analyst used the codes supporting each theme to assign each participant’s responses regarding the first and second halves of practice to one of the emergent themes. For example, if an excerpt from a participant regarding the first half of practice was tagged with *code A*, and *code A* supported *Theme 1*, then the participant’s comments about the first half of practice were assigned to *Theme 1*. Following this, each participant’s questionnaire was reviewed in full to confirm the themes assigned to responses given for the first and second halves of practice, respectively. Where a participant’s responses were ambiguous or multiple codes relating to multiple themes were present, responses to the last two general questions (i.e., questions not referring to a practice half) were used to help inform theme assignment. These procedures were employed to ensure that contextual information was accounted for when assigning themes.

Based on these thematic groupings, we first conducted Fisher’s Exact chi-square tests to determine whether participants’ choice group (SC or YK) was associated with the thematic groups assigned for each half of skill acquisition. Effect sizes for these analyses are represented by Cramer’s *V*, whose value increases from 0 to 1 with the strength of association between two variables. Next, following the analyses employed by Carter et al. (2016) we conducted Mann–Whitney *U* tests for each retention test and views in each half of skill acquisition to compare the dominant Performance view to All other views of haptic assistance. As before, we opted for exact calculations and determined effect sizes as *r*, the ratio of the *z*-score to the square root of the total sample size on which the test-statistic was based (Field, 2009).

### Results

#### Emergent Themes

**FIGURE 5:**
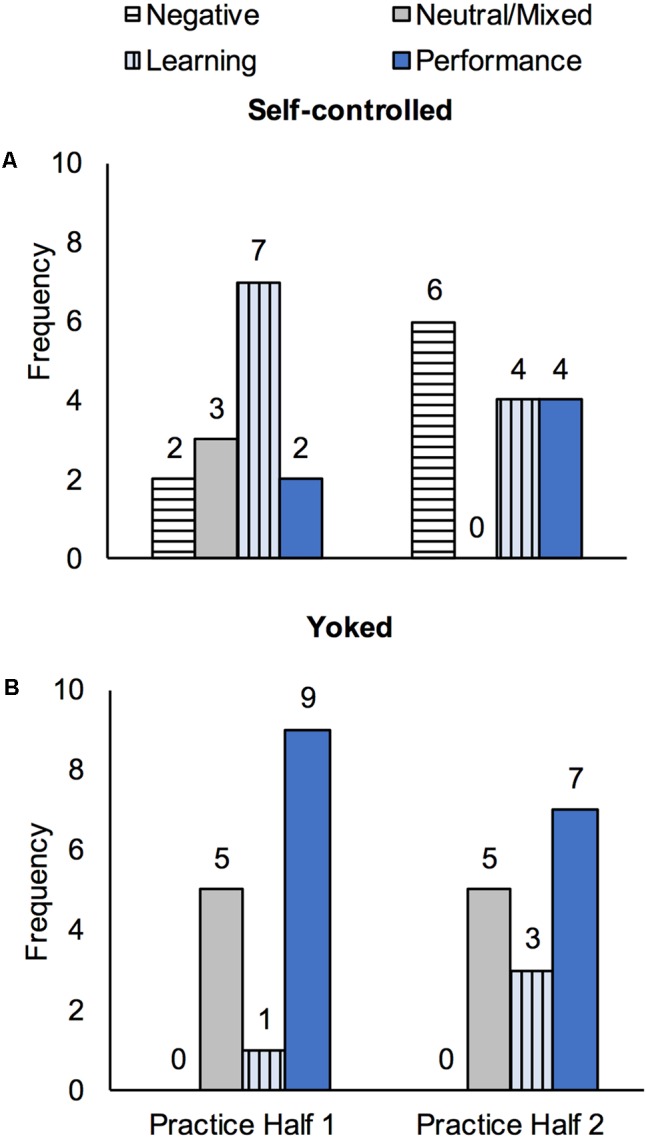
Frequency of each view of haptic assistance (resulting from emergent themes) among Self-controlled **(A)** and Yoked **(B)** participants in each half of skill acquisition.

Analysis revealed four themes related to how SC and YK participants viewed the utility of haptic assistance during skill acquisition: (1) positively for performance; (2) positively for learning; (3) neutrally or heterogeneously with respect to performance and/or learning; and (4) negatively with respect to performance and/or learning. These themes are defined below, the distribution of these views across participants and the two halves of practice are shown in **Figure 5**, and selected quotes are presented in **Table 2**.

**Table 2:** Selected quotes from Self-controlled (SC) and Yoked (YK) participants which support each of the four emergent themes.

**Haptic assistance viewed positively for performance**	
SC	“In B1, I observed there were too many deviations especially toward the end and was taking more time than accounted; so decided to take guidance to improve performance” – P36, 1st half
YK	“It helped decrease time of completion” – P21, 2nd half
**Haptic assistance viewed positively for learning**	
SC	“I wanted to see how much I would improve after using the guidance – in particular how accurate/fast I trace in the trials without guidance after getting the help” – P1, 1st half
YK	“It helped me learn it with assistance first.” – P11, 2nd half
**Haptic assistance viewed neutrally or heterogeneously with respect to performance and/or learning**	
SC	“Only to try it out and see if I could practice after that one experience” – P29, 1st half
YK	“It didn’t matter” – P5, 1st half
**Haptic assistance viewed negatively with respect to performance and/or learning**	
SC	“I wanted the practice to replicate the test conditions so I didn’t use guidance” – P23, 2nd half
YK	None

##### Haptic assistance viewed positively for performance

Participants expressing this view of haptic assistance referred to the use of haptic assistance to facilitate immediate and short-lived changes in the practice experience such as help/assistance, enhanced performance or reduction of effort. Participants expressing this view also spoke of becoming dependent on haptic assistance to maintain their desired level of performance (both accuracy and speed) as well as using haptic assistance to make the task easier, alleviate fatigue and boredom, or reduce the need for concentration or effort. This view was prevalent in the YK group, in both halves of skill acquisition.

##### Haptic assistance viewed positively for learning

Participants expressing this view of haptic assistance referred to the use of haptic assistance to facilitate learning or improve performance on later trials without haptic assistance (e.g., the tests), or otherwise indicated consideration of or aspiration toward ongoing and/or lasting improvement of performance in concert with the use of haptic assistance. This view was primarily observed in the SC group; in fact, it was the dominant view within this group in the first half of practice. Interestingly, whereas the prevalence of this view decreased in the SC group from the first to second half of practice, it increased in the YK group.

##### Haptic assistance viewed neutrally or heterogeneously with respect to performance and/or learning

Participants expressing this view of haptic assistance made mention of it without any clear indication or whether it could or should be used to facilitate performance or learning or indicated multiple views regarding the utility of haptic assistance without a clear dominant view. The comments supporting this theme included descriptions of the chosen haptic assistance schedule, the use of haptic assistance to “test it out” or “try it out” as well as observations that the use of haptic assistance appeared to be unrelated to performance. This theme was more prevalent among the YK group and, interestingly, disappeared from the SC group in the second half of practice.

##### Haptic assistance viewed negatively with respect to performance and/or learning

Participants expressing this view described their refusal of or doubts about the utility of haptic assistance to facilitate performance or learning, or otherwise indicated that haptic assistance would not be beneficial for performance or learning. This theme was notably absent from the YK group and, in the SC group, increased in prevalence from the first to the second half of practice.

#### Relationship between Themes and Choice Groups

For view of haptic assistance in the first half of skill acquisition (refer to **Figure 5** for cell counts), there was a significant association between choice group and first half view, χ^2^(3) = 10.9, *p* = 0.008, Cramer’s *V* = 0.63. This seems to represent the fact that, while 50.0% of SC participants expressed a learning view of haptic assistance, only 6.7% of YK expressed this view; additionally, while 60.0% of YK participants held a performance view, only 14.3% of SC participants did. There was also a significant association between choice group and second half view, χ^2^(3) = 11.9, *p* = 0.005, Cramer’s *V* = 0.64. This relationship seems to represent the fact that while no YK participants expressed negative views of haptic assistance, 42.9% of SC participants did; additionally, while no SC participants expressed neutral or mixed views of haptic assistance, 33.3% of YK participants did.

To have sufficiently large cell sizes for the statistical analysis described below, we selected the dominant view of haptic assistance across all participants (performance view) as a comparator for all other views of haptic assistance. To maintain consistency with that analysis, we repeated the Fisher’s Exact chi-square analyses described above with these two thematic groups (Performance, All other views). These analyses revealed that the association between thematic group and choice group remained significant for the first half of skill acquisition only: first half, χ^2^(1) = 6.4, *p* = 0.021, Cramer’s *V* = 0.47; second half, χ^2^(1) = 1.0, *p* = 0.450, Cramer’s *V* = 0.19.

#### Impact of Views of Haptic Assistance on Learning

**FIGURE 6:**
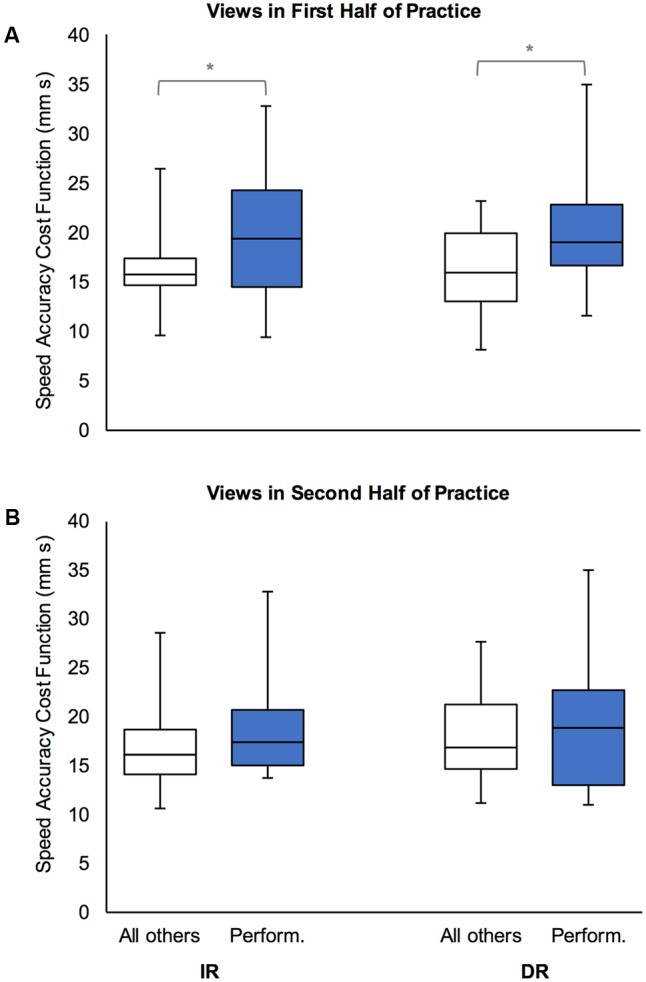
Box and whisker plots of performance efficiency (as measured by the speed accuracy cost function) for each retention test (IR: immediate retention; DR: delayed retention), by views of haptic assistance expressed regarding the first **(A)** and second **(B)** halves of skill acquisition. Views of haptic assistance are indicated by “Perform.” (performance view) or “All others” (all other views: negative, neutral/mixed, and positive for learning). ^∗^*p* < 0.05.

**Figure 6** shows performance on both retentions tests as a function of views of haptic assistance in each half of skill acquisition. Analysis of this data with respect to views of haptic assistance in the first half of practice showed there was a significant effect of view for both the immediate (*U* = 55.00, *z* = -2.0, *p* = 0.049, *r* = -0.37) and delayed retention tests (*U* = 55.00, *z* = -2.0, *p* = 0.049, *r* = -0.37). However, for views of haptic assistance in the second half of practice, there was no effect of view on either the immediate (*U* = 77.00, *z* = -1.0, *p* = 0.340, *r* = -0.18) or delayed retention test (*U* = 95.00, *z* = -0.18, *p* = 0.877, *r* = -0.03).

## Discussion

Participants attempted to learn a self-paced tracing task using a tabletop haptic device under one of three practice conditions: control (no haptic assistance), self-controlled haptic assistance, or yoked haptic assistance. We instructed participants to trace the curve as quickly and as accurately as possible, and measured overall performance efficiency by the speed accuracy cost function where a lower value of the cost function indicates better, overall more efficient task performance. During skill acquisition, the average rate of request for haptic assistance by self-controlled participants (36.3%) was very similar to the 38 and 41% use frequency of assistive devices reported by Hartman (2007) and Chiviacowsky et al. (2012b), respectively. Although there was no difference in the number of haptic assistance requests in the first and second halves of practice, participants improved their performance over the course of skill acquisition. Also, there were no performance differences between groups throughout skill acquisition. Our first hypothesis was that there would be a learning advantage for participants who were able to self-control their use of haptic assistance during skill acquisition. We did not find any direct support for this hypothesis. However, when participants were stratified by frequency of haptic assistance in skill acquisition, as we discovered that the frequency of haptic assistance, as chosen by the SC group, had a significant effect on learning outcomes for the self-controlled group but not the yoked group. Specifically, SC participants who chose a lower frequency of haptic assistance, performed better than those who chose a high frequency on both retention tests. Interestingly, we did find strong support for our second hypothesis that participants’ goals and strategies as determined through qualitative analysis of open-ended questions directly impacted motor learning.

### Self-Control of Haptic Assistance Did Not Directly Impact Motor Learning

While the effect of self-controlled learning has been robust in the motor learning literature, there are a few possible explanations for our failure to find a direct impact of self-control of concurrent haptic assistance for enhancing learning of our tracing task. Firstly, from the perspective of haptic assistance as feedback, Chiviacowsky and Wulf (2005) showed that the benefit of self-control for motor learning was demonstrated when participants were able to make a decision about receiving feedback *after* a trial as opposed to *before* a trial. However, because haptic assistance is provided concurrently, it must be selected before or during a trial. Due to our experimental paradigm, participants chose whether to receive haptic assistance *before* each block of trials. As such, decisions regarding the use of haptic feedback were not based on participants’ performance and this may have limited the extent to which participants engaged with or processed the information as useful feedback. This proposition is supported by evidence in the cognitive skills domain indicating that feedback processing may vary by its utility, that is, the role of feedback in a particular learning task (Arbel et al., 2014).

Secondly, from the perspective of haptic assistance as an assistive device, our task had no appreciable physical risk, e.g., the risk of falling as with previous studies using poles for a ski simulator and stabilometer. Chiviacowsky et al. (2012b) reported that for participants with Parkinson’s disease learning a balance task, self-controlled participants were less nervous than yoked participants before a trial, possibly because having self-controlled access to the physical assistance device relieved anxiety about ability and task performance, which is known to negatively affect motor learning. Although we did not measure anxiety, it is very unlikely that the ability to select haptic assistance had any bearing on our participants’ anxiety levels since the minimal physical risks involved in the task were not differentially affected by the presence of haptic assistance. Furthermore, other studies of self-control of an assistive device have used relatively complex tasks which encouraged participants to use the assistive device to facilitate experimentation with various strategies for performance (Hartman, 2007; Chiviacowsky et al., 2012b) or perform at levels that would not have been possible without more practice (Wulf and Toole, 1999; Wulf et al., 2001), that is, the device facilitated exploring the “perceptual-motor workspace” (Newell, 1991). However, our tracing task was relatively simple and as such, our participants likely did not or could not effectively use the assistance for this type of performance support (Wulf and Shea, 2002).

Lastly, our results were impacted by varying levels of haptic assistance frequency during skill acquisition. Although the effects of the *guidance hypothesis* – the idea that excessive feedback during practice is beneficial for performance but has detrimental effects on learning (Salmoni et al., 1984; Schmidt, 1991) – are well known, we opted to allow participants full control over the feedback schedule. We observed that, similar to a subset of previous findings (Patterson and Carter, 2010; Hansen et al., 2011), participants did not opt for a faded schedule. Instead, participants could be differentiated by whether they choose/experienced a high (>4 of 12 blocks) or low (≤4 of 12 blocks) frequency of haptic assistance. In previous studies of concurrent visual feedback where researchers tried to mitigate the detrimental effects of high feedback frequency, results showed that while reduced-frequency concurrent feedback can be beneficial for performance during skill acquisition, these benefits often disappear on no-feedback retention tests (Park et al., 2000; Camachon et al., 2007). One study using a reduced frequency of haptic assistance was shown to produce learning benefits in comparison to a control group (Marchal-Crespo et al., 2010) but other studies have failed to demonstrate learning benefits in relation to a group that received haptic assistance on all acquisition trials (Marchal-Crespo and Reinkensmeyer, 2008). In contrast, our results showed that reduced-frequency feedback, in relation to high-frequency feedback, showed beneficial performance on no-feedback retention tests, *but* only for self-controlled learners.

### Motor Learning Was Influenced by Frequency and Views of the Utility of Haptic Assistance

Our finding that low frequency haptic assistance was beneficial for SC participants provide some support for the guidance hypothesis (Salmoni et al., 1984; Schmidt, 1991): decisions made by the SC group led to either a high or low frequency of haptic assistance but, unfortunately, a high frequency of haptic assistance is unhelpful at best, if not detrimental, for learning this task (Marchal-Crespo et al., 2010; Williams et al., 2016). Essentially, self-control allowed for strategic (mis)use of haptic assistance and SC participants had the freedom to optimize or sabotage their learning experience. Interestingly, YK participants showed no differences in retention performance based on haptic assistance frequency. This suggests that SC and YK participants may have processed haptic feedback information differently, potentially due to differences in intrinsic motivation, timing of haptic assistance in relation to their current performance, or both. Additional research would be required to confirm information processing differences, isolate the effects of frequency and further explore other related factors (motivation, perceived competence, etc.). While we could have avoided the negative impacts of a high haptic assistance frequency on learning, we were interested to know how participants would choose to use this type of haptic feedback without explicit instructions about its benefits or drawbacks. Knowing that some participants are likely to misuse it in this way opens the door for additional questions about how use frequency might change if participants receive some introductory training or suggestions about “best-practices” for learning. This sort of investigation would mimic the type of control that learners would have under more ecologically valid learning situations such as at-home rehabilitation training or unsupervised simulation-based training for health professions education.

There is evidence that self-control, in and of itself, is not always sufficient to produce a motor learning advantage. For example, Brydges et al. (2009) as well as Zimmerman and Kitsantas (1996) both found that self-controlled learners who set *process* goals outperformed those who set *product or outcome* goals for practicing a wound closure skill using video-based instructions and a dart-throwing task, respectively. In fact, it is widely accepted in the cognitive and academic skills domains that personal factors such as beliefs and knowledge of learning strategies, as well as contextual factors such as the nature of the task and learning environment, can impact the effectiveness of self-controlled learning (Garcia and Pintrich, 1994; Boekaerts and Niemivirta, 2000; Randi and Corno, 2000). Our emergent themes indicated that participants held views or opinions centered around the utility of haptic assistance for performance and learning. In the first half of practice, SC participants were more likely to have a learning view of haptic assistance while YK participants were more likely to have a performance view. This distinction seems to parallel the process vs. outcome goals distinction that can also affect learning outcomes (Zimmerman and Kitsantas, 1996; Brydges et al., 2009). Furthermore, the negative view of haptic assistance was absent from the YK group and we suggest that this offers some evidence of psychological differences between the SC and YK groups. It is possible that less autonomy support for YK participants led them to minimally engage in the learning process and therefore limited how critically they thought about the practice context. In short, the YK participants’ lack of control may have led them to more readily accept all aspects of the training program as good and useful for learning, or to focus on immediate performance instead of strategizing about how to optimize learning (performance on the tests). Further research would be required to explore these ideas.

Ultimately, we propose that SC participants’ views of haptic assistance contributed to their strategic decisions regarding haptic assistance frequency. To test this hypothesis, we ranked the views of haptic assistance, as listed in **Table 2**, from 4 to 1 and conducted Spearman rank correlations between views of haptic assistance in each half of practice and number of guided blocks, for SC and YK groups, respectively. This analysis revealed that for the SC group, the correlation between number of guided blocks and view of haptic assistance in the first half of practice just reached statistical significance, *r*_s_(12) = 0.53, *p* = 0.050, and the correlation was statistically significantly for views in the second half of practice, *r*_s_(12) = 0.81, *p* < 0.001. However, neither correlation was statistically significant for the YK group [both *r*_s_(13) ≤ 0.2, *p* > 0.4]. This means that SC participants who viewed haptic assistance positively were more likely to choose a higher frequency of haptic assistance and suggests that SC participants may have chosen a haptic assistance frequency in accordance with their views about the utility of haptic assistance. Consequently, while self-control may foster specific ways of seeing and interacting with the practice context and these views can have powerful impacts on learning, the strategic decisions borne from these opinions may also modulate learning effects.

The very fact that we could glean such themes from our data is a testament to the utility of open-ended questioning. Earlier studies of self-controlled practice elements for motor learning employed multiple choice questionnaires and focused exclusively on when and why participants chose to receive feedback (Chiviacowsky and Wulf, 2002) or use an assistive device (Hartman, 2007). More recent studies have begun to use open-ended questionnaires (Carter et al., 2016) and interview formats (Laughlin et al., 2015) to allow participants more freedom in their responses. While the findings discussed by Carter et al. (2016) were focused on strategies related to when and why feedback was chosen, Laughlin et al. (2015) discussed more global goals and strategies for performance. Like our findings, these types of global goals and beliefs about the training environment provide key insights into participants’ thought processes and foci of attention during training. Additionally, we have gained information about the participants’ experience of the training system that could be used to inform future studies. For example, some participants mentioned that they would have liked to receive haptic assistance only on the more difficult portions of the curve: “I’d have preferred to receive haptic guidance when I was in the ‘edges’ (corners)” – YK group, P24. This is certainly something that trainers using haptic-based methods could explore. In short, there are substantial benefits to qualitative approaches (in contrast to multiple choice surveys) in motor learning studies when the study goals include gaining in-depth insights into the participants’ rationales and experiences of the practice context.

## Conclusion

In accordance with our first aim, we observed that self-controlled participants chose haptic assistance for about one third of practice blocks but did not employ a faded schedule. Findings with respect to Aims 2 and 3 (determining whether self-control of or opinions about the haptic assistance schedule impacted motor learning) were closely linked. We have provided additional support for the idea that choice alone is not always sufficient to impact motor learning but in fact, that task-relevant choices facilitate or afford access to certain learning strategies or informational benefits that can confer a learning advantage. In the present case, the learning outcomes were impacted by learners’ opinions about the utility of haptic assistance for performance or learning, by way of the haptic assistance frequency chosen during skill acquisition. Self-controlled learners who chose a practice schedule characterized by low haptic assistance frequency (which was associated with more neutral, mixed or negative views about haptic assistance) had a significant learning advantage over self-controlled learners who chose a high haptic assistance frequency (associated with more positive views of haptic assistance for learning and performance). Additionally, our results highlight that instructors should not make assumptions about how participants will view or use elements of the learning environment. In fact, participants’ views or opinions about various training elements will likely be informed by their knowledge of learning strategies and past experiences, and change over the course of practice. If, as movement scientists, the ultimate goal is the implementation of effective real-world training programs, it is important to investigate these assumptions and, if necessary, provide participants with some introductory training or rationale for use of practice elements (Zimmerman and Kitsantas, 1996; Bund and Wiemeyer, 2004; Brydges et al., 2009; Sanli et al., 2013). We invite researchers to continue this line of research by employing rigorous qualitative methods in addition to quantitative evaluations of requests for haptic assistance to further explore how learners enact their views and opinions about the practice context to facilitate motor learning.

## Ethics Statement

This study was carried out in accordance with the recommendations of the University of Toronto Ethics Review Office’s “Guidelines and practices manual for research involving human subjects” with written informed consent from all subjects. All subjects gave written informed consent in accordance with the Declaration of Helsinki. The protocol was approved by the University of Toronto Health Sciences Research Ethics Board.

## Author Contributions

CW and HC were responsible for the conception and design of the work. CW was primarily responsible for data acquisition, analysis and interpretation with contributions from VT for qualitative data analysis and interpretation, and support from HC for interpretation of quantitative data. CW drafted the manuscript while HC and VT provided critical revisions and approved the final version of the manuscript.

## Conflict of Interest Statement

The authors declare that the research was conducted in the absence of any commercial or financial relationships that could be construed as a potential conflict of interest.

## Footnotes

1 There is another perspective through which to understand the relationship between choice and motivation: the self-regulatory strength model (Patall et al., 2008). In this model, the act of self-control requires energy and effort and thus competes with the resources available for participating in an activity or task. Under this model, choices, especially more meaningful ones, could induce a sort of fatigue that can lead to diminished ability to make choices, initiate activities and self-regulate.

2 http://www.brainmapping.org/shared/Edinburgh.php

3 http://www.randomizer.org/
